# A Bayesian Downscaler Model to Estimate Daily PM_2.5_ Levels in the Conterminous US

**DOI:** 10.3390/ijerph15091999

**Published:** 2018-09-13

**Authors:** Yikai Wang, Xuefei Hu, Howard H. Chang, Lance A. Waller, Jessica H. Belle, Yang Liu

**Affiliations:** 1 Department of Biostatistics and Bioinformatics, Rollins School of Public Health, Emory University, Atlanta, GA 30322, USA; johnzon.wyk@gmail.com (Y.W.); howard.chang@emory.edu (H.H.C.); lwaller@emory.edu (L.A.W.); 2 Department of Environmental Health, Rollins School of Public Health, Emory University, Atlanta, GA 30322, USA; xuefeihucn@hotmail.com (X.H.); jessicabelle4@gmail.com (J.H.B.)

**Keywords:** PM_2.5_, Bayesian downscaler, exposure modeling, aerosol optical depth, MODIS

## Abstract

There has been growing interest in extending the coverage of ground particulate matter with aerodynamic diameter ≤ 2.5 μm (PM_2.5_) monitoring networks based on satellite remote sensing data. With broad spatial and temporal coverage, a satellite-based monitoring network has a strong potential to complement the ground monitor system in terms of the spatiotemporal availability of the air quality data. However, most existing calibration models focus on a relatively small spatial domain and cannot be generalized to a national study. In this paper, we proposed a statistically reliable and interpretable national modeling framework based on Bayesian downscaling methods to be applied to the calibration of the daily ground PM_2.5_ concentrations across the conterminous United States using satellite-retrieved aerosol optical depth (AOD) and other ancillary predictors in 2011. Our approach flexibly models the PM_2.5_ versus AOD and the potential related geographical factors varying across the climate regions and yields spatial- and temporal-specific parameters to enhance model interpretability. Moreover, our model accurately predicted the national PM_2.5_ with an *R*^2^ at 70% and generated reliable annual and seasonal PM_2.5_ concentration maps with its SD. Overall, this modeling framework can be applied to national-scale PM_2.5_ exposure assessments and can also quantify the prediction errors.

## 1. Introduction

Particulate air pollution has become a major environmental and public health concern worldwide in recent years. Particularly, particulate matter with aerodynamic diameter ≤2.5 μm (PM_2.5_) is shown to have a strong association with various adverse health outcomes, such as increased mortality and morbidity and aggravated respiratory and cardiovascular symptoms [1]. Ambient PM_2.5_ is either directly emitted from various anthropogenic and biogenic sources or generated in the atmosphere from complex photochemical reactions [2]. Consequently, PM_2.5_ concentrations vary in space and time at sub-kilometer to continental scales [3]. Thus, it is important to accurately assess the population exposure of PM_2.5_. However, in the interest of reducing cost, PM_2.5_ monitors are usually sparsely distributed and tend to be concentrated among urban areas, and most PM_2.5_ monitors operated by the US Environmental Protection Agency (EPA) and the IMPROVE network only operate on a one-in-three-day or one-in-six-day schedule, leaving significant temporal gaps. Due to these spatial and temporal limitations, it is difficult for current PM_2.5_ networks to provide sufficient data to fully assess PM_2.5_ for health effect studies and it could lead to biased results for some key scientific questions.

One emerging solution to these problems is spatial models driven by remotely sensed particle properties from the satellite platform as well as gridded meteorological and land use information. The most robust and widely used satellite parameter is the aerosol optical depth (AOD), which measures the overall particle light extinction caused by airborne particles in the atmospheric column. Many previous studies have shown that AOD has a strong positive association with PM_2.5_. In addition to AOD, previous studies have shown that meteorological and land use information are important factors to predict the ground-level concentration of PM_2.5_ and the relationship between AOD and PM_2.5_ [4,5,6]. All these properties between AOD and PM_2.5_ make it possible to develop statistical methods to calibrate PM_2.5_ using AOD and other geographical factors. Over the past decade, various MODIS-driven PM_2.5_ exposure models have been developed, from relatively simple linear regressions [7] to complex multilevel spatial models [8] and Bayesian hierarchical models [9]. Bayesian hierarchical models have more flexibility in modeling the complex temporal and spatial pattern of PM_2.5_, and compared with other spatial models based on mixed-effects terms [7,8,9,10], one major advantage of the Bayesian model is its underlying nature to quantify the prediction uncertainty through the Markov chain Monte Carlo (MCMC) algorithm, which is crucial for scientific research. Therefore, in this study, we extended the Bayesian model proposed by Chang et al. [9] into a national Bayesian model to examine PM_2.5_ under a national domain.

So far, most satellite-driven PM_2.5_ exposure models have been developed at the urban to regional scales in order to support health effect studies in specific regions [5,9,11,12,13,14,15]. High-performance national scale PM_2.5_ exposure models are still limited partially because of the high-computational demand in order to make national PM_2.5_ prediction surfaces. A couple of national-scale studies involved machine learning methods [16,17]. Di et al. [16] developed a neural network approach, incorporated with convolutional layers to account for spatiotemporal autocorrelation, to predict PM_2.5_ concentrations in the continental United States from 2000 to 2012. Hu et al. [17] developed a random forest model with ~40 predictors to predict PM_2.5_ exposure in the conterminous United States in 2011. These emerging methods can provide relatively high predication accuracy but offer little insight into how different predictors behave across such large domains. For example, random forests only provide an importance value for each predictor to indicate which predictor is more important in the training process. Both neural networks and random forests do not provide quantification of uncertainties in prediction and parameter estimation. These methods also cannot provide straightforward estimates of the model prediction errors. On the other hand, statistical models provide a balance between model predication accuracy and the ability for interpretation and serve as the most reliable and commonly used approaches in calibrating the PM_2.5_. For example, Lee et al. [13] proposed a mixed-effects model with random temporal intercept and slope on AOD to evaluate the time-varying effects. This type of model assumes that the temporal-random-effect-based model requires the independence assumption between different days, which is generally not practical and, thus, they have limited power to make predictions out of the temporal domain. They also fail to adjust the spatial variability in large spatial domains and can provide biased results. Similar for the hierarchical models [5,11,12], it is tricky to quantify the uncertainties in prediction or parameter estimation based on such models, which limits their power to be used in real applications. Thus, all these models are not directly applicable to the national domain.

Chang et al. [9] reported a Bayesian downscaling model which adopted the Gaussian spatial process to incorporate the spatial correlation into the model, which increases the power to borrow information across neighborhoods, through which the challenge of spatial misalignment between the point-referenced monitoring measurements and the gridded areal AOD data can be solved. It also models the conditional correlation between adjacent observed days, which allows us to estimate the random effects on the day without PM_2.5_ measurements. In addition, this model adopts a full Bayesian approach, by which the model uncertainty can be obtained easily. However, this model is only applicable to small spatial domains for three reasons. First, it assumes that the temporal correlation structure is constant across the whole spatial domain, but based on our study, this is not realistic in a large spatial domain. Similarly, it assumes that the spatial correlation structure is constant across the whole year, which is not realistic. Second, the original model is not flexible enough to capture the huge spatial variability in the national domain. For example, it assumes a constant effect of land use across different states that fails to consider the localized difference, which is one of the goals of a national study. Finally, directly generalizing the original model is computationally expensive because the spatial correlation matrix is of high dimensions and is very sparse.

In this paper, we enhanced and expanded the original Bayesian downscaler to the entire continental United States We developed a regional- and temporal-specific Bayesian downscaling approach to gain more flexibility. Our model incorporated AOD data, meteorological fields, and land use variables to estimate daily ground-level PM_2.5_ concentrations over the conterminous United States for the year 2011. The estimated regional- and temporal-specific parameters were scientifically meaningful and the prediction accuracy was evaluated through general and spatial cross-validation frameworks. Our model predicted the daily averaged PM_2.5_ concentrations across the entire continental United States and also the prediction uncertainty maps.

## 2. Data and Methods

### 2.1. Data Collection

The 24-h averaged PM_2.5_ measurements for 2011 were downloaded from the US EPA’s Air Quality System Technology Transfer Network (http://www.epa.gov/ttn/airs/airsaqs/). Collection 6 level 2 Aqua MODIS retrievals at a nominal spatial resolution of 10 km were regridded to the 12 × 12 km^2^ Community Multi-Scale Air Quality (CMAQ) modeling system (https://www.epa.gov/cmaq). Regridding to a fixed grid is necessary when modeling with level 2 MODIS data because MODIS pixels shift in location and size with each satellite overpass. In addition, given the 10-km nominal resolution of MODIS AOD pixels, regridding to the commonly used 12-km CMAQ grid does not compromise the AOD spatial resolution significantly. Doing so may also benefit future comparisons between CMAQ simulation results and our model predictions. We calculated AOD averages using AOD retrievals from the combined deep-blue and dark-target parameters. Meteorological fields were obtained from the North American Regional Reanalysis (NARR) (http://www.emc.ncep.noaa.gov/mmb/rreanl/), with a spatial resolution of ~32 km and a temporal resolution of 3 h, and the North American Land Data Assimilation System Phase 2 (NLDAS-2) (http://www.emc.ncep.noaa.gov/mmb/rreanl/), with a spatial resolution of ~13 km and a temporal resolution of 1 h. Elevation data at a spatial resolution of ~30 m were downloaded from the National Elevation Dataset (http://ned.usgs.gov). Road data were extracted from ESRI StreetMap USA. Percentage forest cover data at a spatial resolution of ~30 m were extracted from the 2011 Landsat-derived land cover map downloaded from the National Land Cover Database (NLCD) (http://www.mrlc.gov). Primary PM_2.5_ emissions were obtained from the 2011 EPA National Emissions Inventory (NEI) facility emissions report (https://www.epa.gov/air-emissions-inventories/2011-national-emissions-inventory-nei-data).

### 2.2. Climate Regions and Temporal Domains

**Figure 1:**
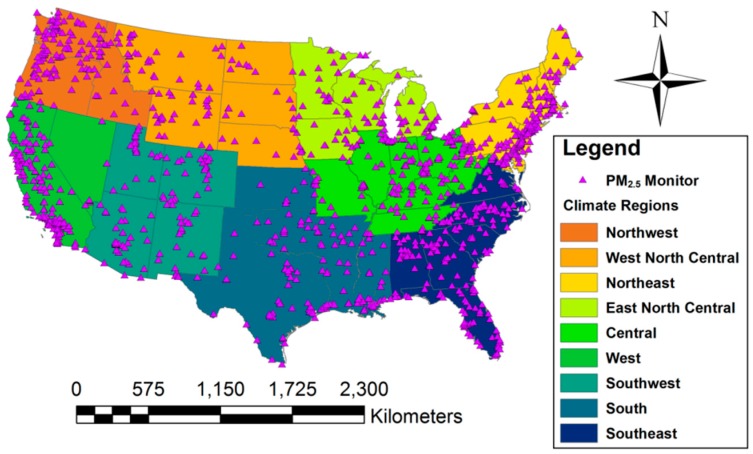
The nine climate regions and the spatial location of the monitors.

To improve computational efficiency, we divided the conterminous U.S. into nine NOAA-defined climate regions [18], which include Northeast, Southeast, South, Ohio Valley (Central), Upper Midwest (East North Central), Northern Rockies and Plains (West North Central), Southwest, Northwest, and West (Figure 1). After examining aerosol light extinction measurements in various regions of the world, Anderson et al. [3] reported that the typical mesoscale variability of lower-tropospheric particles ranges between 40 and 400 km Therefore, by dividing our national domain into nine multistate regions, we were still able to sufficiently capture the spatial and temporal correlations of ground-level PM_2.5_. We added a 100-km buffer to each climate region and averaged overlapping predictions from neighboring regions to generate a smooth national PM_2.5_ concentration surface. In addition, the spatial pattern varies significantly across the year. To reduce the high computational demand of our Bayesian model, we divided the year 2011 into three 4-month temporal periods and developed a Bayesian downscaling model in each period. Since the typical PM_2.5_ residence time in the boundary layer ranges from a couple of days to two weeks, this treatment had minimal impact on model performance.

### 2.3. National Bayesian Downscaling Model

For each regional and temporal subdomain, we adopted the basic framework of the Bayesian downscaling model proposed by Chang et al. [9]. In this model, let *PM(s,t)* denote the PM_2.5_ concentration at location s and day t, where s can be viewed as the unique spatial coordinates. Similarly, let *AOD(s,t)* denote the AOD measurement at the grid cell containing the monitor s and day t. For one specific climate region reg, a function of s and the temporal domain, the first level model between AOD and PM_2.5_ is given as

(1) $$PM\left(s,t\right)=\alpha_{0}\left(s,t\right)\;+\;\alpha_{1}\left(s,t\right)\;AOD\left(s,t\right)\;+\gamma_{reg,\;tem}\left(s,t\right)\;Z\left(s,t\right)\;+\varepsilon \left(s,t\right)$$

where *α*_0_*(s,t)* and *α*_1_*(s,t)* are the day-specific and location-specific random intercept and slope and the residual error *ε*(s,t) is assumed to be independently normal with mean zero and regional- and temporal-specific variance *σ*_reg,tem_^2^. *Z(s,t)* represents for the covariates having a constant association with PM_2.5_, where γ_reg,tem_ represents for the regional- and temporal-specific fixed effect between *Z(s,t)* and *PM(s,t)*. Here, *Z(s,t)* includes fire, forest coverage, emission, relative humidity (RH), temperature, wind speeds, major roadway length, boundary layer height (Hpbl), and the interaction between AOD and temperature.

The spatiotemporal random effects *α*_0_*(s,t)* and *α*_1_*(s,t)* are specified using additive setting. For clarity, we present the model setting for one specific region and temporal domain: *α_i_(s,t)* = *β_i_*(s) + *β_i_*(t), *i* = 0,1, where *β_i_*(s) and *β_i_*(t) are independent spatial and temporal effects. The spatial effects are modeled using a latent structure of two independent spatial Gaussian processes W_1_(s) and W_2_(s), where *β*_0_(s) = c_1_W_1_(s) and *β*_1_(s) = c_2_W_1_(s) + c_3_W_2_(s) and the covariance function of W*_i_*(s) for each region is given by the exponential function multiplied by a tapering function. The regional-specific temporal effects *β*_0_(t) and *β*_1_(t) are modeled as two independent daily time series using a first-order random walk, which can be defined through the conditional distribution of a particular day given all other days. More details about the model specifications can be found in the online supplementary materials.

### 2.4. Model Fitting and Prediction

Model fitting was carried out using Markov chain Monte Carlo (MCMC) techniques [9,19]. Details of the MCMC algorithms and the prior settings can be found in the online supplementary materials. Prediction performance was evaluated using two different cross-validation methods: fully random cross-validation (random CV) and spatial cross-validation (spatial CV) [9,20]. In random CV, we randomly split the data into 10 folds and fit the model using 9 folds and evaluated the fitted model using the remaining fold, which can be used to evaluate the overall prediction ability of our approach. The spatial CV was similar to random CV except that rather than randomly splitting the data, we split the data based on its spatial location. The spatial CV results were used to evaluate the ability in spatial extrapolation. In addition, through the MCMC approach, we quantified the prediction uncertainty, i.e., interval estimates. We also calculated the prediction statistics by comparing the predicted PM_2.5_ measurements with the observations, which include root-mean-square error (RMSE), 90% posterior interval (PI) length, and its empirical coverage probability and linear coefficient of determination *R*^2^ value. All analyses were carried out in R version 3.2.3 (https://www.r-project.org/).

## 3. Results

### 3.1. Data Description and Summary

**Figure 2:**
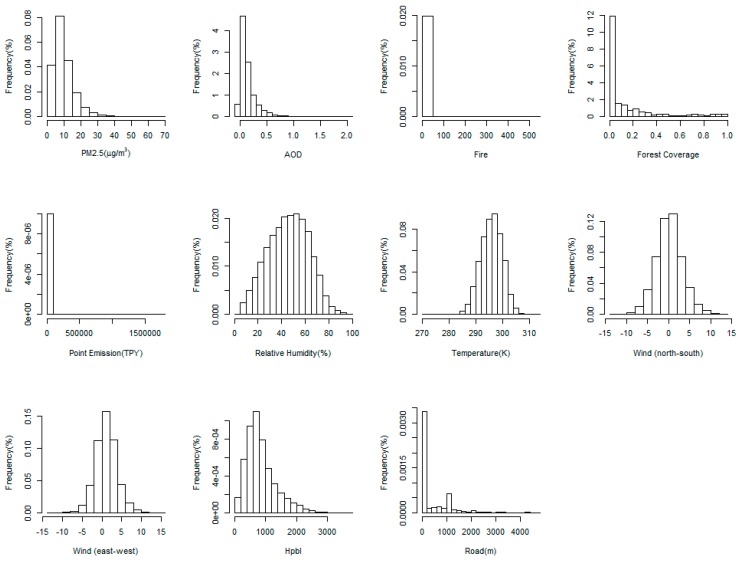
Histograms of the dependent and independent variables.

The histograms of all variables are illustrated in Figure 2, which shows that all the variables are approximately unimodal and log-normal distributed. Log-transformation was conducted for fire, emission, and road for the following analysis. Z-transformation was conducted for all variables except for PM_2.5_ and AOD to remove the collinearity between covariates and to make the scale comparable. The annual mean PM_2.5_ concentration for all monitors was 9.88 μg/m^3^, with an SD of 6.17 μg/m^3^. The overall mean of AOD was 0.14, with an SD of 0.15. The region-specific descriptive statistics for PM_2.5_ and AOD are summarized in Table 1. The number of records, monitors, days, and the percentage of data coverage for each region are summarized in Table 2. Among the nine regions, the Ohio Valley has the highest mean PM_2.5_ concentration at 11.29 μg/m^3^ and it has most records (18,642) and monitors (361). In terms of missing AOD data, the West has the best data coverage (30%), while the Northeast has the worst (12%) due to both cloud cover and snow cover in winter.

### 3.2. Regional and Temporal Varying Geographical Associations

In this section, we explore the different patterns across climate regions and temporal domains revealed by the significant parameters in our model. First of all, the national Bayesian downscaling model fits well across different regions and temporal domains in terms of model *R*^2^ and slope, as shown in Table 3. The Northeast tends to have the best model fitting. The climate regions with the highest set of *R*^2^ are the Upper Midwest (0.85) and Northeast (0.84) regions. The slopes in these two climate regions are consistently higher than 0.95 across all time domains, indicating minimal systematic biases in model fitting. On the other hand, the model tends to have a lower *R*^2^ in the South and Northwest regions, where the annual *R*^2^ for the South climate region is 0.64 and the annual *R*^2^ for the Northwest climate region is 0.63.

Table 3 presents all the significant geographical and meteorological factors (*p*-value < 0.05), which exhibit substantial inter-region differences among the regional models. First of all, AOD is the most important covariate and is significant for most regions and temporal domains. We noticed that the effect of AOD on PM_2.5_ is weaker in May through August than other months. This pattern is commonly observed in all climate regions after we condition the temperature to be the average level in the specific spatial and temporal domain. Furthermore, fire, RH, temperature (TMP), and the interaction between AOD and TMP are significant across all regions. Other covariates, including forest coverage, emission, wind speed, Hpbl, and road length, vary across regions and temporal domains. Forest coverage is a significant factor in explaining the pattern of PM_2.5_ in the West, Northwest, and Southwest climate regions across the entire year but is not significant in the Northern Rockies and Plains regions. Emission is not significant in most regions except the West, where it significantly explains the variability of PM_2.5_. On the other hand, Hpbl has a significantly negative association with PM_2.5_ in the West region but is not significant in the Northwest and Northeast regions.

### 3.3. Model Cross-Validation

**Figure 3:**
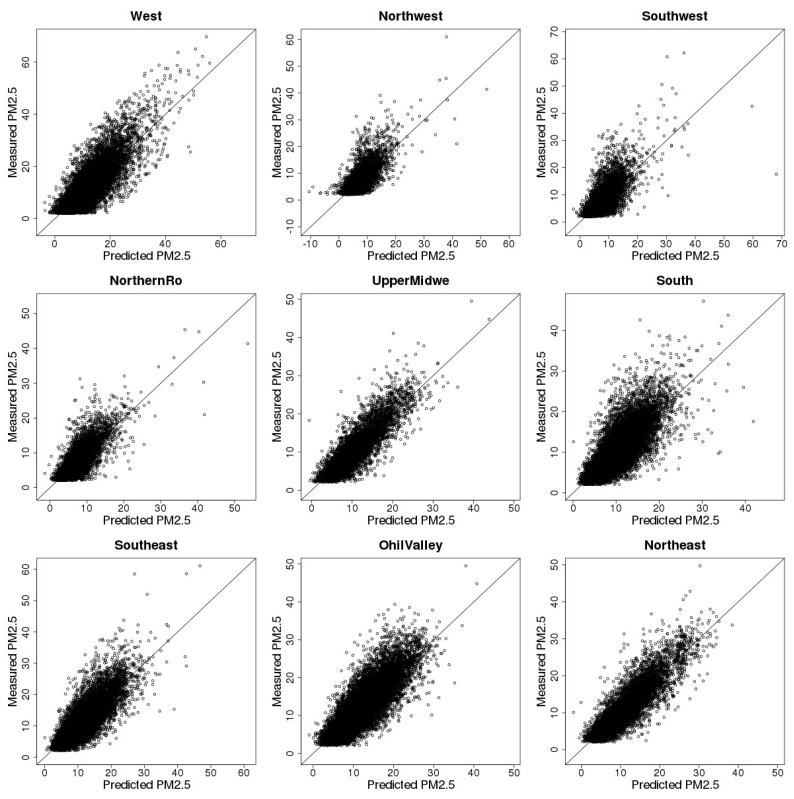
Tenfold cross validation results.

**Figure 4:**
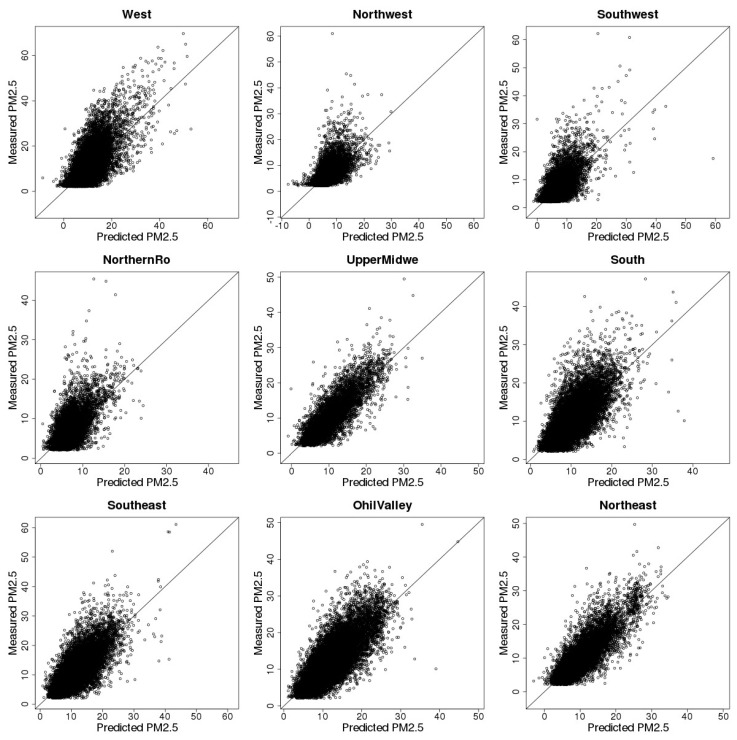
Spatial 10-fold cross validation results.

The overall CV *R*^2^ for the entire study area and study period is 0.70 and the slope between predicted PM_2.5_ and the observed PM_2.5_ is 0.98, indicating good agreement between CV estimates and observations. Regional results of random and spatial 10-fold CV including *R*^2^ and slope are listed in Table 4 and Table 5. Results show that the CV-based performance of our model varies across regions. For example, our model achieves the highest *R*^2^ under both CV settings (random *R*^2^ = 0.78, spatial *R*^2^ = 0.70) in the Northwest as well as in the Upper Midwest and Ohio Valley regions. On the other hand, the Southwest region has the lowest *R*^2^ of 0.54 for random CV. For spatial CV, our model does not perform as well as for random CV, where in the Northwest region, the random CV *R*^2^ is 0.60 and the spatial CV *R*^2^ is only 0.39. Specifically, Figure 3 and Figure 4 show the scatterplots of CV estimates and observed PM_2.5_ concentration levels across nine climate regions. The CV-based PM_2.5_ estimates have good linear agreement with the observations in the West, South, Upper Midwest, Southwest, Northwest, and Ohio Valley regions. However, in the Southwest, North, and Northwest regions, the model tends to underestimate at higher PM_2.5_ concentrations.

### 3.4. Model Prediction

**Figure 5:**
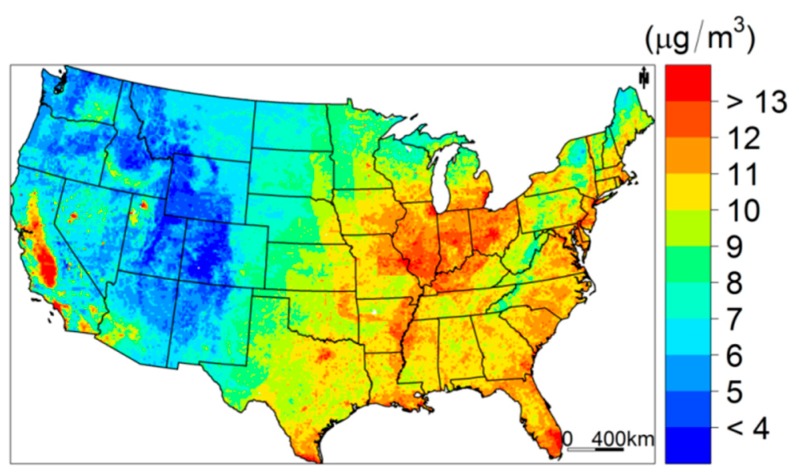
Predicted annual PM_2.5_ concentration across the continental United States.

**Figure 6:**
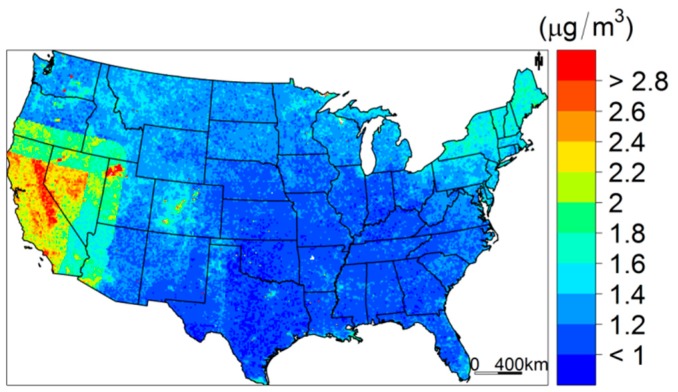
The uncertainty (standard deviation) of the predicted annual PM_2.5_ concentration across the continental United States.

The predicted annual average PM_2.5_ concentrations and their model-based SDs are visualized in Figure 5 and Figure 6. As shown in Figure 5, the predicted annual mean of PM_2.5_ concentration is smoothed across all the spatial domains, even among the climate buffer regions, indicating that the national Bayesian model fits the data well. Furthermore, a strong spatial differential pattern exists in the annual PM_2.5_ spread, where the PM_2.5_ concentration is higher in eastern regions than western regions. California, the Great Lakes regions, and the east coast regions, including New York and Washington, have especially high annual average PM_2.5_ concentrations. On the other hand, the lowest annual PM_2.5_ concentration is found in Midwestern states, such as Utah, Colorado, Wyoming, and Idaho, with extensive forest coverage and sparse human activities. These indicate that our model can capture large-scale spatial patterns of PM_2.5_ well. Our model is also able to discover the small features of the predicted PM_2.5_ concentration surface, where we can observe high PM_2.5_ concentration levels in urban centers such as Atlanta, Dallas, Houston, Miami, and Salt Lake City.

Regarding prediction uncertainty, Figure 6 shows the spatial spread of the standard deviation of the annual average PM_2.5_ concentrations. The West region, including California and Nevada, has a higher SD compared with other regions. The South and Southeast regions have the lowest SD on average. More specifically, from the spatial distribution of SD, we observed a higher peak at the Miami, Houston, and Dallas areas and Colorado state. Similarly, we visualized the predicted seasonal average PM_2.5_ concentrations and their SDs and the results are in Supplemental Figures S1 and S2.

## 4. Discussion

In our national Bayesian downscaling approach, we first adopted nine climate regions and three temporal regions to separate the data into subregions, which provided more flexible model fitting. Then, we utilized the Bayesian downscaling approaches in each sub-block to quantify the geographical patterns and the association between AOD and PM_2.5_. Compared with regional models, including regional hierarchical models, mixed-effects models, and regional Bayesian downscaling methods, our approach provides nationally cohesive predictions and quantifies the model prediction errors. Compared with machine learning models (e.g., neural networks and random forests), our approach incorporates the core of the statistical approaches, providing insights into the physical and geographical information of the problem. The model uncertainty provided by the Bayesian approach is much more informative than that generated from machine learning models.

Our approach has several strengths. First, it uses a latent spatial process to incorporate spatial correlation, which can borrow information across neighborhoods and is able to make more reliable predictions compared with mixed-effects models. Second, based on the climate region and temporal separation, our model is much more flexible in terms of model fitting and therefore fits the data better than the traditional Bayesian models. As shown in the Table 1, there is a significant difference in the geographical patterns across regions and temporal domains, which is an important sign that in different climate regions, the association between AOD and PM_2.5_ is complex and region specific. This further confirms that using a single model for a whole national domain is not realistic, as it cannot reveal the real physical mechanism in which researchers are interested. Moreover, our proposed approach can perform parallel setting and is much faster than traditional approaches.

Finally, compared with the machine learning method for national calibration, our approach has slightly weaker prediction ability, probably because of the fewer predictors used in our models than in theirs. For example, Di et al. [16] included more than 50 predictors in the neural network model, and the random forest model in Hu et al. [17] contained ~40 predictors. In addition, both approaches used convolutional layers for nearby PM_2.5_ measurements and land use terms in their models, and both studies point out that convolutional layers can help to improve prediction accuracy. Although we could have included these predictors in our models, it would have required additional computing resources and consumed additional computing time. We will address this issue in future research.

## 5. Conclusions

We presented a national Bayesian downscaler model to estimate daily PM_2.5_ concentrations in the continental United States using satellite aerosol remote sensing data and meteorological and land use parameters. Overall, our national Bayesian downscaling model performs well at the national scale. It has the advantage of explicitly displaying the important predictors of PM_2.5_ in different geographical regions, which allows model simplification and further improvements of model performance. It should be noted that the goal of this approach is to study the geographical patterns across different regions and seasons and our approach successfully provides great insights into these and provides much more informative results than machine learning methods. As we mentioned, the limited prediction ability of our approach in some specific regions, i.e., the South region, is one limitation. The reason for this is that even though we separated the national domain into sub-blocks, each region is still very large, which makes it difficult for a single model to fit across such a large domain. Thus, one future direction of this model is to provide a more flexible approach.

## Figures and Tables

**Table 1:** Descriptive statistics for PM_2.5_ and aerosol optical depth (AOD).

Regions	PM_2.5_ (SD)	AOD (SD)
West	10.72 (7.17)	0.10 (0.12)
Northwest	6.23 (4.05)	0.12 (0.11)
Southwest	7.40 (4.75)	0.10 (0.11)
Northern Rockies and Plains	7.40 (4.11)	0.12 (0.13)
Upper Midwest	10.33 (5.87)	0.18 (0.17)
South	10.17 (5.09)	0.13 (0.15)
Southeast	10.83 (5.34)	0.15 (0.17)
Ohio Valley	11.29 (5.79)	0.17 (0.17)
Northeast	10.68 (6.10)	0.19 (0.19)

**Table 2:** Region-specific counts and data coverage.

Regions	Number of Records	Number of Days	Number of Monitors	Coverage
West	17,096	356	159	30%
Northwest	9486	295	170	19%
Southwest	9567	363	138	19%
Northern Rockies and Plains	7463	328	150	15%
Upper Midwest	6208	304	145	14%
South	15,899	364	189	23%
Southeast	17,525	361	257	19%
Ohio Valley	18,642	354	361	15%
Northeast	8913	302	238	12%

**Table 3:** Statistically significant geographical and meteorological predictors.

Region	Temporal	AOD	Fire	Forest	Emission	RH	TMP	Vgrd	Ugrd	Hpbl	Road	AOD * TMP	*R* ^2^	Slope
West	1	21.2 (5.9)		−0.7 (0.3)	0.6 (0.2)	2.5 (0.2)	2.4 (0.4)	1.2 (0.2)		−0.2 (0.1)		9.8 (3.4)	0.65	0.88
	2	4.1 (1)		−0.8 (0.3)	0.4 (0.1)	0.7 (0.1)	2.7 (0.2)			−0.1 (0.1)			0.77	0.94
	3	31.2 (5.2)	0.2 (0.1)	−1.9 (0.3)	0.8 (0.3)	0.6 (0.1)		1.4 (0.1)	−0.4 (0.1)	−0.7 (0.1)		−8.5 (2.5)	0.72	0.91
Northwest	1			−1.5 (0.5)	−0.7 (0.3)	−1.9 (0.5)							0.57	0.84
	2	5.4 (1.1)	0.1 (0)	−0.2 (0.1)		0.4 (0.1)	1.5 (0.2)					4.4 (1)	0.62	0.92
	3	25.4 (3.8)	0.4 (0.1)	−0.4 (0.2)			1.2 (0.4)	−0.4 (0.1)				14.7 (2.1)	0.69	0.9
Southwest	1	10.6 (5)	0.3 (0.1)	−0.7 (0.2)		0.5 (0.2)	2.4 (0.3)	0.5 (0.1)				−11 (2.8)	0.69	0.89
	2	5.5 (1.8)		−0.3 (0.1)		0.4 (0.2)	3.5 (0.3)	0.3 (0.1)	0.6 (0.1)				0.6	0.88
	3	18.8 (4.5)		−0.5 (0.2)			0.7 (0.2)		−0.2 (0.1)	−0.3 (0.1)			0.68	0.9
Northern Rockies and Plains	1		0.4 (0.1)			1.2 (0.3)		0.7 (0.2)					0.82	0.95
	2	4.4 (1.5)	0.3 (0.1)			0.3 (0.1)	2.4 (0.2)	0.4 (0.1)				3.1 (1)	0.67	0.92
	3	11.1 (2.1)	0.3 (0.1)				2.1 (0.2)		−0.6 (0.1)	−0.4 (0.1)			0.73	0.92
Upper Midwest	1					0.5 (0.3)		1.3 (0.2)		−0.4 (0.2)			0.79	0.95
	2	4.4 (1.7)	0.3 (0.1)	−0.6 (0.2)		0.9 (0.1)	2.7 (0.3)	1 (0.1)	−0.3 (0.1)			3.7 (1)	0.82	0.95
	3	9.5 (3)	0.4 (0.1)	−0.6 (0.2)	0.3 (0.1)		2.5 (0.2)	0.4 (0.1)	−0.3 (0.1)	−0.2 (0.1)			0.85	0.96
South	1	13.1 (2.2)	0.5 (0)	−0.3 (0.1)			1.4 (0.2)	0.3 (0.1)	−0.2 (0.1)				0.59	0.91
	2		0.2 (0.1)			0.5 (0.1)	4.2 (0.3)	−0.2 (0.1)	0.2 (0.1)		0.3 (0.1)	4.5 (1.1)	0.67	0.94
	3	14.7 (1.9)	0.3 (0)	−0.7 (0.1)		−0.2 (0.1)	1 (0.2)	0.3 (0.1)	−0.4 (0.1)	−0.4 (0.1)	0.3 (0.1)		0.65	0.93
Southeast	1	15.1 (1.9)	0.3 (0)	−0.3 (0.1)		−0.3 (0.1)	0.8 (0.2)	0.5 (0.1)				4.6 (0.9)	0.68	0.94
	2	4.6 (1.6)	0.1 (0)			1.7 (0.2)	7 (0.4)	−0.6 (0.1)	0.2 (0.1)			6.1 (1.1)	0.74	0.95
	3	11.1 (1.4)	0.3 (0)	−0.6 (0.1)		−0.7 (0.1)	0.8 (0.2)	0.7 (0.1)		−0.3 (0.1)		6.9 (1.1)	0.69	0.94
Ohio Valley	1	21.2 (2.9)	0.7 (0)	−0.5 (0.1)		0.4 (0.1)	0.7 (0.2)	0.7 (0.1)	−0.3 (0.1)			5.5 (1)	0.68	0.94
	2	5.7 (1.3)				2.2 (0.1)	5.5 (0.3)	0.3 (0.1)			0.2 (0.1)	2.9 (0.7)	0.74	0.95
	3	14.4 (5.2)	0.3 (0.1)	−0.8 (0.1)			1.7(0.2)	0.5 (0)	−0.3 (0)	−0.3 (0.1)		3.3(1.3)	0.77	0.95
Northeast	1	10.6 (2.8)	0.4 (0.1)					0.9 (0.1)	−0.4 (0.1)				0.8	0.95
	2		−0.2 (0.1)			1.2 (0.2)	6.4 (0.4)	−0.4 (0.1)				8.5 (1.1)	0.84	0.96
	3	31 (2.5)	1.5 (0.2)	−0.8 (0.2)		1.8 (0.2)	1.4 (0.4)					27.5 (2)	0.8	0.95

* All predictors are significant at *α* = 0.05 level.

**Table 4:** Tenfold cross validation results.

Regions	*R* ^2^	Intercept	Slope
West	0.69	0.04	0.99
Northwest	0.60	0.35	0.95
Southwest	0.54	0.40	0.94
Northern Rockies and Plains	0.60	0.29	0.95
Upper Midwest	0.76	−0.04	0.99
South	0.59	0.27	0.97
Southeast	0.69	0.19	0.98
Ohio Valley	0.71	0.07	0.99
Northeast	0.78	0.07	0.99

**Table 5:** Spatial 10-fold cross validation results.

Regions	*R* ^2^	Intercept	Slope
West	0.46	0.36	1.02
Northwest	0.39	1.01	0.83
Southwest	0.40	0.96	0.87
Northern Rockies and Plains	0.37	0.94	0.90
Upper Midwest	0.69	−0.01	0.99
South	0.50	0.38	0.96
Southeast	0.58	0.77	0.92
Ohio Valley	0.65	0.18	0.97
Northeast	0.70	0.33	0.97

## Supplementary Materials

The following are available online at http://www.mdpi.com/1660-4601/15/9/1999/s1, Figure S1: Seasonal Predicted PM_2.5_ Concentration, Figure S2. Standard Deviation of Seasonal Predicted PM_2.5_ Concentration, Text S1: Detailed Model Specification and Estimation Procedure.
