# Supplementary figures and images for: A Bayesian Downscaler Model to Estimate Daily PM2.5 Levels in the Conterminous US
Source: Int J Environ Res Public Health. 2018 Sep 13;15(9):1999. doi: 10.3390/ijerph15091999 (PMC6164266; doi:10.3390/ijerph15091999)

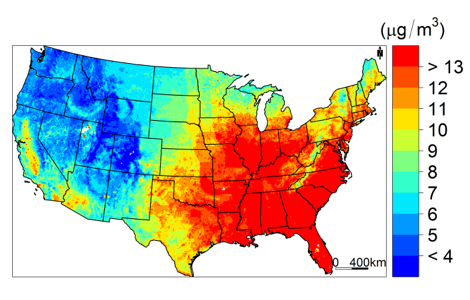

Supplement: Supplementary file 1 [file ijerph-15-01999-s001.zip › s1_2.png]

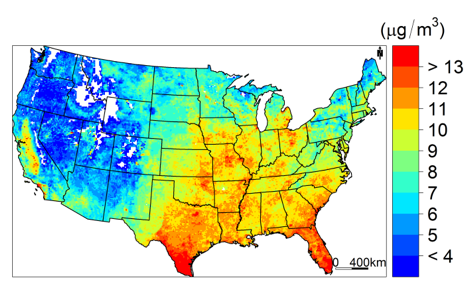

Supplement: Supplementary file 1 [file ijerph-15-01999-s001.zip › s1_1.png]

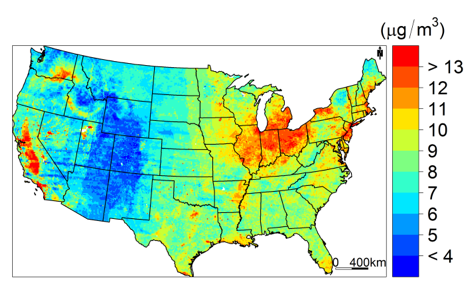

Supplement: Supplementary file 1 [file ijerph-15-01999-s001.zip › s1_3.png]

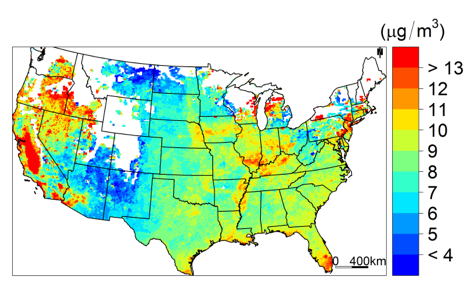

Supplement: Supplementary file 1 [file ijerph-15-01999-s001.zip › s1_4.png]

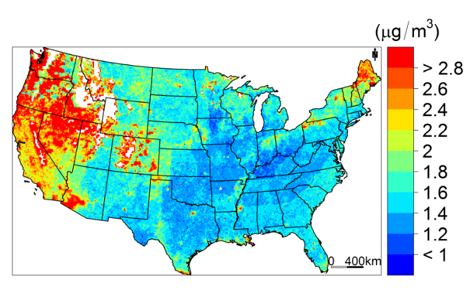

Supplement: Supplementary file 1 [file ijerph-15-01999-s001.zip › s2_1.png]

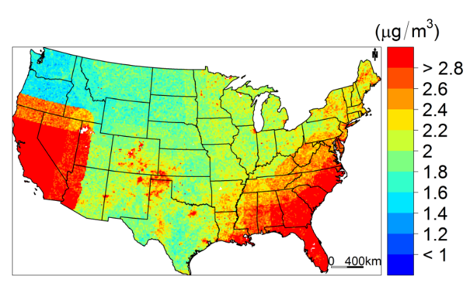

Supplement: Supplementary file 1 [file ijerph-15-01999-s001.zip › s2_2.png]

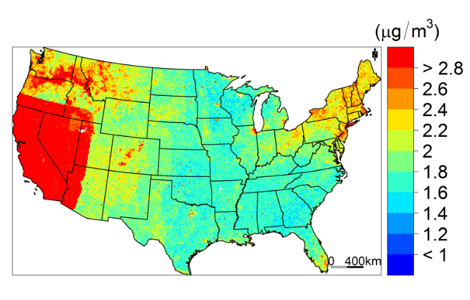

Supplement: Supplementary file 1 [file ijerph-15-01999-s001.zip › s2_3.png]

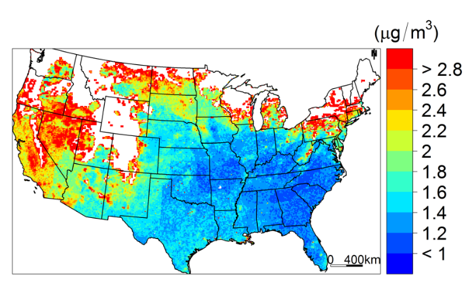

Supplement: Supplementary file 1 [file ijerph-15-01999-s001.zip › s2_4.png]
